# Comparative Study of M(Ⅱ)Al (M=Co, Ni) Layered Double Hydroxides for Silicone Foam: Characterization, Flame Retardancy, and Smoke Suppression

**DOI:** 10.3390/ijms231911049

**Published:** 2022-09-21

**Authors:** Lin-Lin Zhou, Wen-Xiong Li, Hai-Bo Zhao, Bin Zhao

**Affiliations:** 1 Institute of Functional Textiles and Advanced Materials, Engineering Research Center for Advanced Fire-Safety Materials Development and Applications, College of Textiles & Clothing, Qingdao University, 308 Ningxia Road, Qingdao 266071, China; 2 Collaborative Innovation Center for Eco-Friendly and Fire-Safety Polymeric Materials (MoE), National Engineering Laboratory of Eco-Friendly Polymeric Materials (Sichuan), College of Chemistry, Sichuan University, Chengdu 610064, China

**Keywords:** silicone foam, layered double hydroxides, transition metal, flame retardancy, smoke suppression

## Abstract

To compare the different actions of the two representative transition metal cations of Co^2+^ and Ni^2+^ in layered double hydroxides (LDHs), CoAl-LDH and NiAl-LDH intercalated with CO_3_^2−^ were synthesized, and the chemical structures, microstructures, and surface areas thereof were successfully characterized. Then, the two LDHs were utilized as flame retardants and smoke suppressants for silicone foam (SiF). The densities, flame retardancy, smoke suppression, thermal stabilities, and compressive strengths of the two SiF/LDHs nanocomposites were investigated. The introduction of LDHs slightly decreased the density of SiF due to the catalytic actions of Co and Ni during the foaming process of SiF. With respect to the flame retardancy, the addition of only 1 phr of either CoAl-LDH or NiAl-LDH could effectively improve the limiting oxygen index of SiF from 28.7 to 29.6%. Based on the results of vertical flame testing and a cone calorimeter test, the flame retardancy and fire safety of the SiF were effectively enhanced by the incorporation of LDHs. In addition, owing to the good catalytic action and large specific surface area (NiAl-LDH: 174.57 m^2^ g^−1^; CoAl-LDH: 51.47 m^2^ g^−1^), NiAl-LDH revealed higher efficiencies of flame retardancy and smoke suppression than those of CoAl-LDH. According to the results of energy-dispersive X-ray spectroscopy, Co and Ni participated in the formation of protective char layers, which inhibited the release of SiO_2_ into the gas phase. Finally, the influences on the thermal decomposition and compressive strength for SiF resulting from the addition of LDHs are discussed.

## 1. Introduction

In recent years, due to their natural abundance, non-toxicity, and large specific surface area, layered double hydroxides (LDHs) have garnered extensive attention [1]. LDHs, which are known as anionic clays, consist of brucite-like metal ion layers, interlayer water molecules, and anions. The general formula of LDHs can be labeled as [M^2+^_1−x_M^3+^_x_(OH)_2_]^x+^(A^n−^)_x/n_·mH_2_O, in which M^2+^ and M^3+^ denote divalent and trivalent metal cations, respectively, and A^n−^ represents interlayer anions [2,3,4,5]. There are many kinds of strategies for fabricating LDHs, such as co-precipitation [6,7,8], hydrothermal synthesis [9,10], the urea hydrolysis method [11,12], microencapsulation technology [13], reconstruction methods [14,15], and mechano-chemical methods [16,17,18]. Among them, the co-precipitation method is the simplest and most commonly used, and it can be used to directly synthesize LDHs with a variety of inorganic and organic interlayer anions, obtaining highly tunable properties of the structure and function of LDHs [19].

The functionalization of LDHs can be achieved by adjusting the types and proportions of metal cations and the types and quantities of interlayer anions [1]. The highly tunable properties of their structure and function make LDHs a hot research topic, and LDH-based functional composites have been widely applied in many fields, including those of catalysts [20,21,22], adsorbents [23,24,25], supercapacitors [26,27,28,29], biomedicine [30,31], and flame retardants [2,32,33]. In an important application, LDHs have been considered as inorganic flame-retardant fillers and synergists due to their moderate toxicity and low cost [34]. Compared to other metal-modified flame retardants, LDHs are metal-containing fillers without a traditional flame-retardant element [35,36,37]. The classical LDH flame retardants are MgAl-LDH and ZnAl-LDH. At present, LDHs containing transition metals, such as CoAl-LDH, FeZn-LDH, CuAl-LDH, and CoNi-LDH, are also commonly used as flame retardants due to their special catalytic effects [11,37,38,39,40]. Jaerger et al. [41]. intercalated CoAl-LDH, NiAl-LDH, and ZnAl-LDH with distinct organic anions as fillers for low-density polyethylene and evaluated their thermal stability and flammability. The addition of CoAl-LDH and NiAl-LDH did not affect the thermal stabilities of the nanocomposites, but ZnAl-LDH decreased the thermal stability of the samples; the flame-retardant effects of NiAl-LDH and ZnAl-LDH were better than that of CoAl-LDH. This demonstrates that the types of metal cations in LDHs have different effects on the flame retardancy and thermal stability of the nanocomposites. Nagendra et al. [42] used ultrasound to delaminate CoAl-LDH, ZnAl-LDH, and CoZnAl-LDH in order to form LDH nanoparticles of a smaller size, and the delaminated LDH solution was utilized to fabricate highly dispersed isotactic polypropylene. The results showed improvements in the flame retardancy and thermal stability of the nanocomposites in the following order: CoZnAl-LDH > ZnAl-LDH > CoAl-LDH. As a result, the exhibition of desirable properties in polymer/LDH nanocomposites depends on the selection of the metal constituents of the LDHs.

Silicone foam (SiF) is an organic–inorganic hybrid polymer elastomer foam with excellent and comprehensive properties, and it has the advantages of silicone rubber and foam materials. It is widely used in aerospace, the construction industry, medical equipment, and other fields [43]. Compared to some traditional polymeric foams, such as polystyrene foam, polyurethane foam, and organic aerogel materials [44,45,46,47], SiF shows good flame retardancy due to its special siloxane structure. However, it is still necessary to improve the flame retardancy and smoke suppression of SiF due to its many organic groups and unique cell structure [48]. There are various methods for improving the flame retardancy and smoke suppression of SiF, such as base rubber modification, surface treatment, and addition of flame-retardant fillers [49]. Among them, adding flame-retardant fillers method is relatively simple and has a low cost. However, the high loadings and low flame-retardant efficiency limit its application [43,50,51,52]. In our previous work [53], the addition of transition-metal-containing NiTi-LDH was able to significantly improve the flame retardancy and smoke suppression of SiF. The incorporation of only 1 phr NiTi-LDH endowed SiF with a high limiting oxygen index (LOI) of 30.3% and a UL-94 V-0 level. Moreover, the total smoke production of the silicone foam could be reduced by 73%. Thus, LDHs containing transition metals might contribute to the enhancement of the fire safety of SiF.

In this work, CoAl-LDH and NiAl-LDH were facilely prepared through a co-precipitation process and used as flame retardants and smoke suppressants in silicone foam. The influences of the existence of Co and Ni and the physical structures of the LDHs on the representative properties of the SiF/LDH nanocomposites were examined and discussed according to the results for the LOI, vertical flame testing, cone calorimeter test, thermogravimetric analysis, and compression performance test.

## 2. Results and Discussion

### 2.1. Characterization of CoAl-LDH and NiAl-LDH

**Figure 1:**
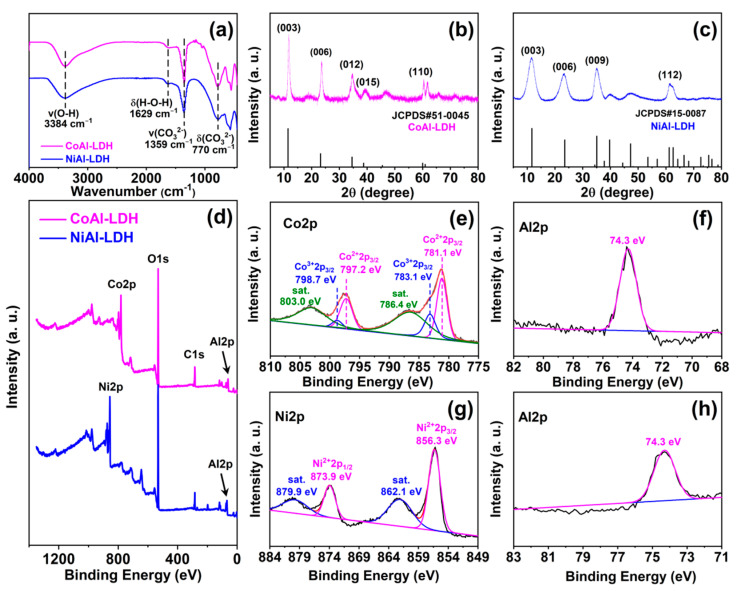
(**a**) FTIR spectra of CoAl-LDH and NiAl-LDH; XRD patterns of (**b**) CoAl-LDH and (**c**) NiAl-LDH; (**d**) XPS total survey spectra of CoAl-LDH and NiAl-LDH; (**e**) Co2p and (**f**) Al2p spectra of CoAl-LDH; (**g**) Ni2p and (**h**) Al2p spectra of NiAl-LDH.

By using a facile co-precipitation method at an ambient temperature, CoAl-LDH and NiAl-LDH were successfully synthesized with intercalated CO_3_^2−^. Figure 1a displays the FTIR spectra of the CoAl-LDH and NiAl-LDH. As with typical LDHs, the IR bands centered at 1629 and 3384 cm^−1^ were assigned to the O—H bending and stretching vibration peaks, respectively, which resulted from the interlayer water molecules and hydroxyl groups in the hydrotalcite layers [54]. The characteristic peaks at 770 and 1359 cm^−1^ belonged to the stretching and bending modes of CO_3_^2−^, respectively, indicating the presence of CO_3_^2−^ in the interlayer of CoAl-LDH and NiAl-LDH [55].

The XRD patterns (Figure 1b,c) were recorded to confirm the phase composition and crystallinity of the CoAl-LDH and NiAl-LDH nanosheets. The two diffractograms exhibited characteristic diffraction patterns of layered materials, showing peaks at (003) and (006), which matched well with the standard LDH lamellar structures (JCPDS X-ray powder diffraction files 51-0045 and 15-0087 are indexed). The diffraction planes of (003) and (006) of CoAl-LDH at 11.75° and 23.60° corresponded to lattice spacings of 0.753 and 0.375 nm, respectively. For NiAl-LDH, the lattice spacings corresponding to *d_(003)_* (2θ = 11.62°) and *d_(006)_* (2θ = 23.25°) peaks were 0.765 and 0.384 nm, respectively. Moreover, the LDH structure could be classified as rhombohedral with a = 0.3064 and c = 2.2573 nm as refined lattice parameters [56,57]. Combined with the results of FTIR and XRD, it could be demonstrated that carbonate-intercalated CoAl-LDH and NiAl-LDH were successfully synthesized.

Figure 1d presents the XPS survey spectra of CoAl-LDH and NiAl-LDH, and the typical elements in the two LDHs were detected. For CoAl-LDH, as shown in Figure 1e, the Co^2+^ (781.1 and 792.2 eV) and Co^3+^ (783.1 and 798.7 eV) signals appeared in the Co2p spectrum, and the content ratio of Co^2+^/Co^3+^ was 2.579 [29]. The characteristic peak at 74.3 eV could be attributed to Al2p, confirming the existence of Al^3+^ in the CoAl-LDH [58]. As shown in the high-resolution spectrum of Ni2p for NiAl-LDH (Figure 1g), the two characteristic peaks at 856.3 and 873.9 eV were assigned to Ni2p_3/2_ and Ni2p_1/2_, respectively, and were accompanied by two characteristic satellite peaks at 862.1 and 879.9 eV [59]. Additionally, the Al2p spectrum (Figure 1h) confirmed the existence of Al^3+^ (74.3 eV) in the NiAl-LDH [58]. The chemical formulas of the LDHs were further determined by using the ICP-OES test, and the corresponding data are summarized in Table 1; the Co/Al molar ratio was 2.32 and the Ni/Al molar ratio was 2.28. Combining the content ratio of Co^2+^/Co^3+^ in the XPS test and the general formula of the LDHs, the chemical composition of CoAl-LDH was obtained as Co^2+^_0.504_Co^3+^_0.195_Al_0.301_(OH)_2_(CO_3_^2−^)_0.248_. Similarly, it could be concluded that the chemical composition of NiAl-LDH was [Ni_0.695_Al_0.305_(OH)_2_(CO_3_^2−^)_0.153_].

**Figure 2:**
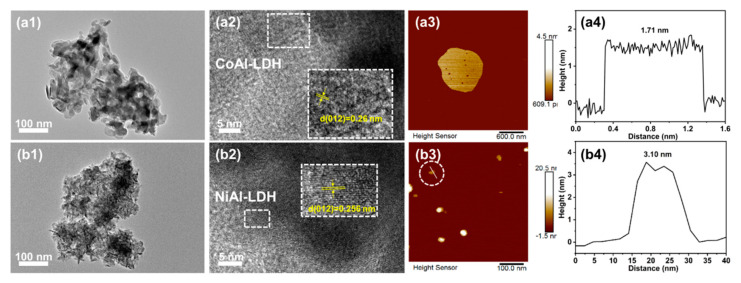
TEM and HRTEM images of CoAl-LDH (**a1**,**a2**) and NiAl-LDH (**b1**,**b2**); AFM images and the corresponding height profiles of CoAl-LDH (**a3**,**a4**) and NiAl-LDH (**b3**,**b4**).

The TEM images of CoAl-LDH and NiAl-LDH presented in Figure 2a1,b1 show the typical platelet-like morphology of the LDHs. As observed in the HRTEM images shown in Figure 2a2,b2, the lattice spacing of 0.26 nm corresponding to the (012) lattice planes was detected in the XRD patterns of the CoAl-LDH and NiAl-LDH. Furthermore, the thicknesses of CoAl-LDH and NiAl-LDH were measured by using the cross-sections of the corresponding scanned lines in the AFM images. As illustrated in Figure 2a3,b3, the thicknesses of CoAl-LDH and NiAl-LDH were determined to be 1.71 and 3.10 nm, respectively, and they were approximately 2–4 times thicker than the interlayer spacing obtained in the XRD test. This can be ascribed to a slight layer accumulation phenomenon occurring in LDHs [60].

**Figure 3:**
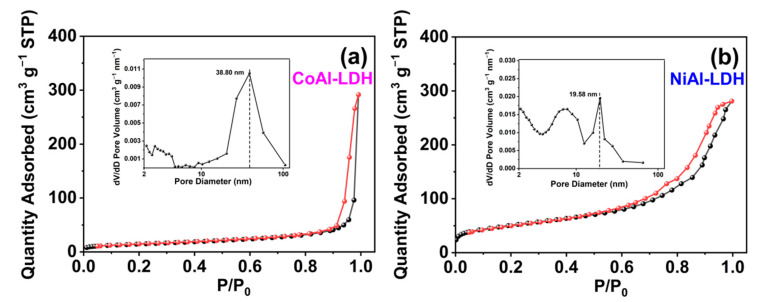
N_2_ adsorption–desorption isotherms of (**a**) CoAl-LDH and (**b**) NiAl-LDH and the corresponding pore diameter distribution curves (insets).

The surface areas and pore size distributions of CoAl-LDH and NiAl-LDH were further characterized by with Brunauer–Emmett–Teller (BET) N_2_-sorption measurement. Figure 3a,b show the N_2_ adsorption–desorption isotherms and pore diameter distribution curves of CoAl-LDH and NiAl-LDH, respectively. Both CoAl-LDH and NiAl-LDH exhibited typical IV isotherms, implying the existence of a mesoporous pore structure [61,62]. The BET specific surface area of NiAl-LDH was 174.57 m^2^ g^−1^, which was larger than the value of 51.47 m^2^ g^−1^ for CoAl-LDH. The Barrett–Joyner–Halenda (BJH) method was used to estimate the pore diameter distributions of the samples. The average pore diameters of CoAl-LDH and NiAl-LDH according to the BJH method were, respectively, 34.66 and 10.42 nm.

### 2.2. Morphological Analysis of the SiF/LDH Nanocomposites

**Figure 4:**
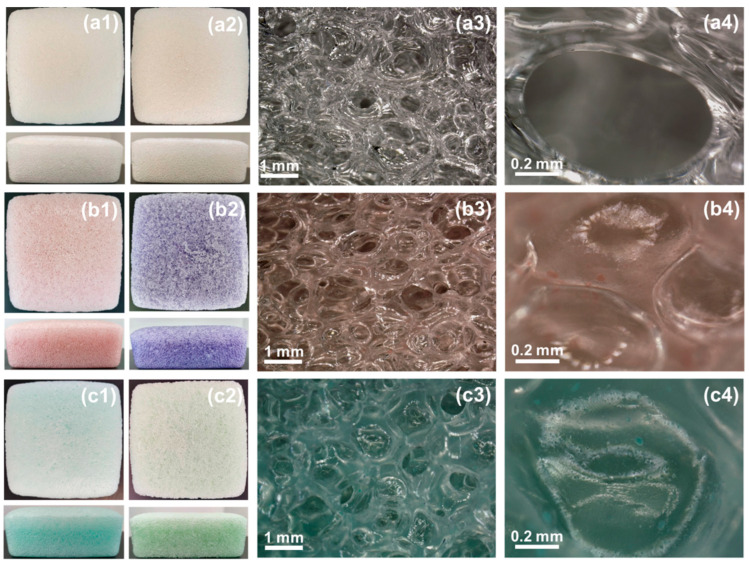
Photographs of the SiF/LDH nanocomposites before (**a1**,**b1**,**c1**) and after (**a2**,**b2**,**c2**) thermal treatment at 150 °C for 2 h (“13 cm × 13 cm” corresponding to the “length × wide” of the foams here). Microscopic surface topographies of the SiF/LDH nanocomposites (**a3**,**a4**,**b3**,**b4**,**c3**,**c4**).

The photographs and microscopic surface topographies of the SiF nanocomposites are shown in Figure 4. From changed colors in the photographs of the SiF/LDH nanocomposites before and after thermal treatment can be ascribed to the heat transition of the LDHs. The microscopic surface topographies of the SiF/LDH nanocomposites were given by an ultra depth-of-field microscope. In this way, we could more intuitively observe the cellular structures of the silicon foam so as to explore the effect of the addition of the LDHs on the cell structure of the silicone foam. As shown in the microscopic surface topographies of SiF/LDHs nanocomposites, the cells of SiF/CoAl-LDH-1.0 were smaller than those of the untreated silicone foam, while the cells of SiF/NiAl-LDH-1.0 were slightly changed. It could be observed that the LDHs were uniformly dispersed in the bubble hole walls of the SiF/LDH nanocomposites. As shown in Table 2, the densities of SiF/CoAl-LDH-1.0 and SiF/NiAl-LDH-1.0 were 0.27 and 0.26 g cm^−3^, which were close to that of untreated SiF (0.28 g cm^−3^). Thus, the addition of the two LDHs had little influence on the density of the silicone foam. The slightly decreased densities of the SiF/LDH nanocomposites could be ascribed to the fact that Co and Ni have the same catalytic effect as that of Pt catalysts, thus increasing the cross-linking speed of SiF.

### 2.3. Flame Retardancy and Fire Behavior of the SiF/LDH Nanocomposites

**Figure 5:**
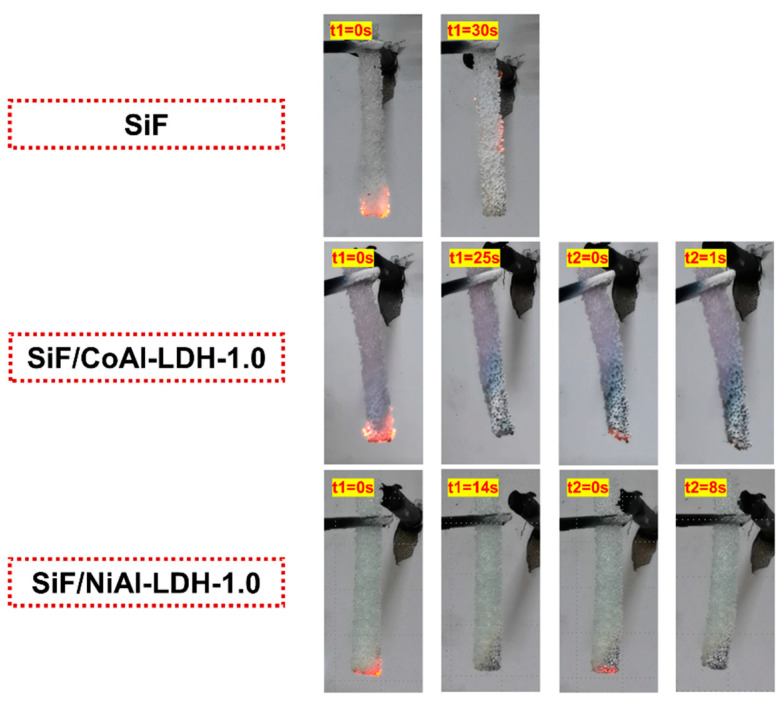
Video snapshots of the SiF/LDH nanocomposites during vertical flame testing.

The flame retardancy of the SiF/LDH nanocomposites was assessed through vertical flame testing and the limited oxygen index, the results of which are provided in Table 2 and Figure 5. After incorporating only 1 phr of CoAl-LDH and NiAl-LDH, the LOI value of SiF was increased from 28.7% to 29.6%. As shown in Figure 5, SiF continued to burn beyond 30 s after ignition and showed no rating in the vertical flame testing. Fortunately, both 1.0 phr CoAl-LDH and NiAl-LDH were able to endow SiF with a UL-94 V-1 rating. The two LDHs endowed SiF with the same LOI value and the same rating of vertical flame testing. However, as shown in Figure 5, the SiF/NiAl-LDH-1.0 sample was damaged less than the SiF/CoAl-LDH-1.0 sample, implying a better flame resistance.

**Figure 6:**
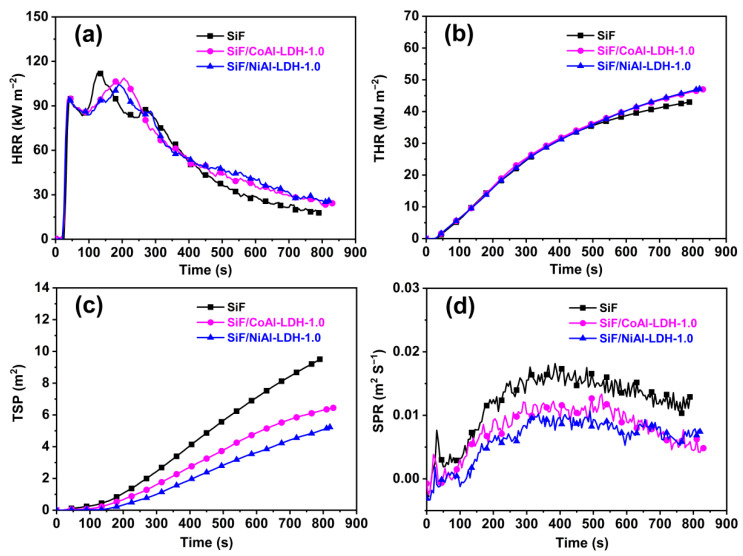
Typical curves derived from the cone calorimeter tests: (**a**) HRR, (**b**)THR, (**c**) TSP, and (**d**) SPR curves.

The cone calorimeter test can be used to evaluate the fire behaviors of polymeric materials at a bench scale [63], and it provides an abundance of important parameters, including the peak of heat-release rate (pHRR), total heat release (THR), time to pHRR (t_p_), total smoke release (TSR), char residue, and fire-growth rate (FIGRA, defined as the maximum of HRR(t)/t [64]). After evaluating the fire behaviors, the typical curves of the SiF/LDH nanocomposites were plotted, as shown in Figure 6, and the detailed characteristic data are provided in Table 3. As can be observed in the HRR and THR curves, CoAl-LDH and NiAl-LDH had limitations in reducing the heat release of silicone foams, reflecting a slight improvement in flame retardancy under this forced radiation fire testing. However, the addition of each kind of the two LDHs was able to effectively delay the time to pHRR from 130 to 205 s (SiF/CoAl-LDH-1.0) and 190 s (SiF/NiAl-LDH-1.0), indicating a delayed time to flashover of SiF during burning. Thus, the derived value of the FIGRA parameter of the SiF was lowered from 0.87 to 0.53 and 0.55 kW m^−2^·s^−1^ by the introduction of both CoAl-LDH and NiAl-LDH, respectively, leading to an enhanced fire safety of SiF. More importantly, as in the previous report [53], the LDHs exhibited a high efficiency in smoke suppression. The TSP values of SiF/CoAl-LDH-1.0 and SiF/NiAl-LDH-1.0 declined by 33% and 45%, respectively, from the value of 9.5 m^2^ for the untreated SiF. As shown in Figure 6d, both CoAl-LDH and NiAl-LDH endowed the SiF with a remarkable reduction in the smoke production rate with loadings of only 1 phr. In addition, NiAl-LDH exhibited a better smoke suppression effect than that of CoAl-LDH in the SiF. In addition to the good catalytic action of Ni, the better smoke suppression performance of NiAl-LDH might depend on its larger specific surface area (NiAl-LDH: 174.57 m^2^ g^−1^; CoAl-LDH: 51.47 m^2^ g^−1^), as it can absorb a large amount of smoke.

### 2.4. Char Residue Analyses

**Figure 7:**
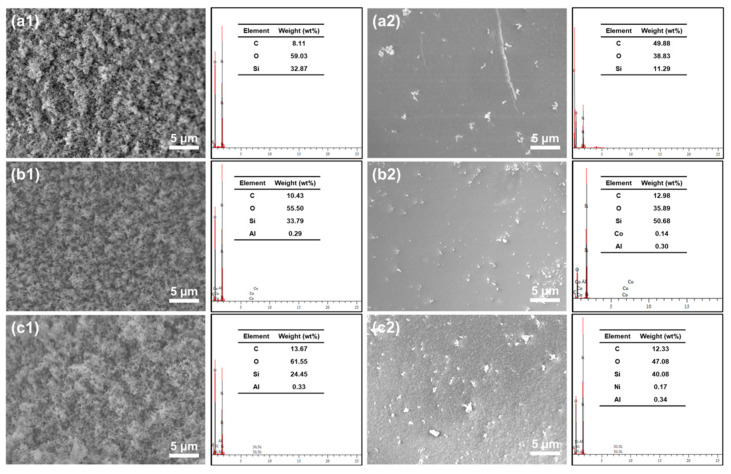
SEM-EDS images of the outer surface (SiF: **a1**; SiF/CoAl-LDH-1.0: **b1**; SiF/NiAl-LDH-1.0: **c1**) and inner surface (SiF: **a2**; SiF/CoAl-LDH-1.0: **b2**; SiF/NiAl-LDH-1.0: **c2**) of char residues from the nanocomposites after vertical flame testing.

The outer surface and inner surface of char residues from the SiF/LDH nanocomposites were characterized through SEM-EDS, as illustrated in Figure 7. The outer char surfaces of the SiF and SiF/LDH nanocomposites were rough and cellular. The outer char surfaces of SiF/CoAl-LDH-1.0 and SiF/NiAl-LDH-1.0 were more compact than that of the untreated SiF. Cracks were observed on the inner char surface of SiF, while the SiF/LDH nanocomposites retained an intact char layer covered with significantly white particles. According to the EDS analyses, Co and Al elements were detected in the inner char layer of SiF/CoAl-LDH-1.0 and Ni and Al were detected in that of SiF/NiAl-LDH-1.0, indicating that both CoAl-LDH and NiAl-LDH contributed to the formation of the inner char layer. In addition, the content of Si in the outer char layer of the SiF was higher than that in the inner char layer, which may have been due to the volatilization of gas-phase SiO_2_ through the outer char layer. After adding the two LDHs, the content of Si in the inner char layer increased, implying that the LDHs could inhibit the release of SiO_2_ into the gas phase. The char layer played the role of a physical barrier and showed better protective effects for the foam matrix.

### 2.5. Thermal Decomposition of CoAl-LDH, NiAl-LDH, and the SiF/LDH Nanocomposites

**Figure 8:**
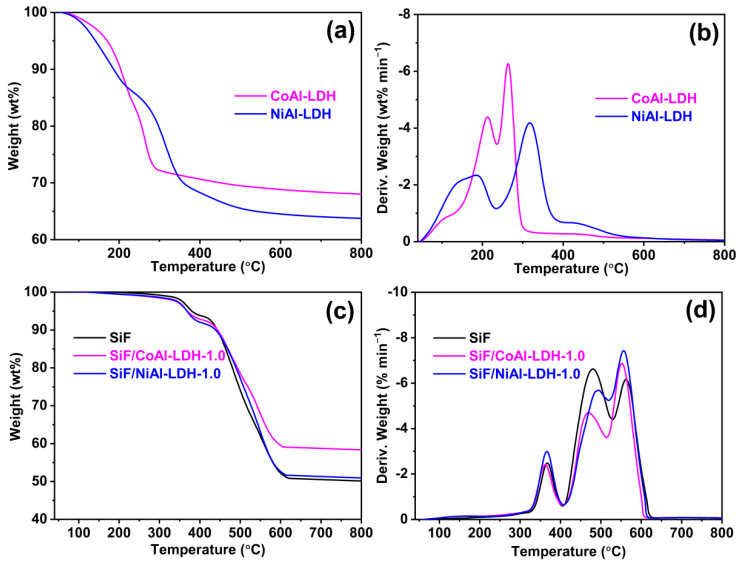
TGA curves for the (**a**,**b**) CoAl-LDH, NiAl-LDH, and (**c**,**d**) SiF/LDH nanocomposites in an air atmosphere.

The TG and DTG curves of the CoAl-LDH, NiAl-LDH, and SiF/LDH nanocomposites were recorded in an air atmosphere. As shown in Figure 8 and Table 4, both of the LDHs displayed two stages of weight-loss processes. Before 240 °C, the first stage took place due to the removal of surface-adsorbed water and interlayer water. The second weight-loss stage (CoAl-LDH: 240–300 °C; NiAl-LDH: 240–400 °C) could be mainly attributed to the complete dehydroxylation process of the LDH layers and the removal of the intercalated CO_3_^2−^ [65,66]. CoAl-LDH started to decompose at 171 °C, while NiAl-LDH started to decompose at 141 °C. Meanwhile, the amount of char residue of CoAl-LDH at 800 °C was 68.0 wt% greater than that of NiAl-LDH (63.7 wt%). The above results indicate that CoAl-LDH showed a higher thermal stability and char-foaming ability than those of NiAl-LDH. The SiF/LDH nanocomposites were prepared by adding CoAl-LDH or NiAl-LDH to the silicone foam. As a result of oxidation degradation of the methyl group in the silicone side chains, the SiF decomposed at 380 °C under air [67]. With the increase in temperature, further decomposition of SiF occurred at 480 and 562 °C, which was attributed to the random scission reaction of the polymer backbone and the unzipping of the Si-OH end-groups to generate cyclic oligomers [68]. Compared with SiF, the initial decomposition temperature of the SiF/LDH nanocomposites decreased by 11–12 °C, showing a lower initial thermal stability than that of SiF. However, the addition of CoAl-LDH was able to significantly improve the char residue rate of silicone foam at 800 °C due to the large amount of char residue of CoAl-LDH at high temperatures.

### 2.6. Mechanical Properties of the SiF/LDH Nanocomposites

**Figure 9:**
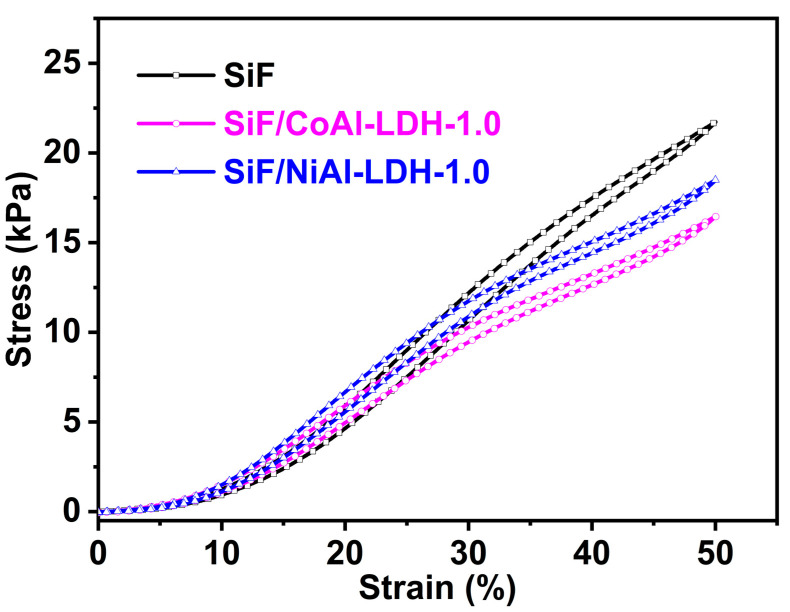
The compressive stress–strain curves of the SiF and SiF/LDH nanocomposites.

The compressive stress–strain curves of the SiF/LDH nanocomposites are shown in Figure 9; the compressive strength of the SiF was 21.6 ± 1.2 kPa. Compared with SiF, the compressive strengths of the SiF/CoAl-LDH and SiF/NiAl-LDH nanocomposites decreased to 16.7 ± 2.0 and 18.6 ± 1.0 kPa, respectively. The reduction in the compressive strengths can be attributed to be the changed morphologies and decreased densities of the SiF/LDH nanocomposites. As previously described, in detail, the cell sizes of SiF/CoAl-LDH-1.0 and SiF/NiAl-LDH-1.0 increased compared with that of SiF; the density of the SiF/LDH nanocomposites was lower than that of SiF.

## 3. Materials and Methods

### 3.1. Materials

Anhydrous sodium carbonate and anhydrous ethanol were provided by Sinopharm Chemical Reagent Co., Ltd. (Shanghai, China). Cobalt nitrate hexahydrate (Co(NO_3_)_2_·6H_2_O, 99%, cas. 10026-22-9), sodium hydroxide (NaOH, 97%, cas. 1310-73-2), and aluminum nitrate nonahydrate (Al(NO_3_)_3_·9H_2_O, 99%, cas. 7784-27-2) were purchased from Shanghai Aladdin Biochemical Technology Co., Ltd. (Shanghai, China). Nickel nitrate hexahydrate (Ni(NO_3_)_2_·6H_2_O, 97%, cas. 13478-00-7) was purchased from Tianjin Heowns Opde Technologies Co., Ltd. (Tianjin, China). Hydroxyl-terminated polydimethylsiloxane (HO-PDMS, 3000 mPa·s) was provided by Jiashan Jiangnan Textile Material Co., Ltd. (Suzhou, China). Karstedt-type catalyst (Pt, CAT-PL-56, 5000 ppm) and polymethylhydrogensiloxane oil with 1.5% active hydrogen content (High-H PMHS, XiameterTM MHX 1107) were, respectively, purchased from Shin-Etsu Chemical Co., Ltd. (Tokyo, Japan) and Dow Corning Co., Ltd. (Midland, MI, USA). α,ω-divinylpolydimethylsiloxane (Vi-PDMS, 10,000 mPa·s) and polymethylhydrogen silicone oil with an active hydrogen content of 0.1% (Low-H PMHS, DY-H212) were bought from Shandong Dayi Chemical Co., Ltd. (Yantai, China). As an inhibitor, 2,4,6,8-tetramethyl-2,4,6,8-tetravinylcyclotetrasiloxane (V4) was purchased from Guangdong Wengjiang Chemical Reagent Co., Ltd. (Shaoguan, China).

### 3.2. Synthesis of CoAl-LDH and NiAl-LDH

CoAl-LDH and NiAl-LDH were synthesized by using co-precipitation methods under similar conditions. In detail, 0.01 mol Al(NO_3_)_3_·9H_2_O was mixed with 0.02 mol Co(NO_3_)_2_·6H_2_O or Ni(NO_3_)_2_·6H_2_O and dissolved in 80 mL of deionized water. Then, Na_2_CO_3_ solution (20 mL, 0.1 mol L^−1^) was introduced into the aforementioned mixed salt solution under magnetic stirring. Afterwards, the pH of the mixture was adjusted to 11.2 (for the CoAl-LDH system) or 8.8 (for the NiAl-LDH system) by using aqueous NaOH (1 mol·L^−1^) [69]. After reacting for 6 h, the suspension was aged for another 15 h at room temperature. The precipitates were filtered and washed using deionized water and ethanol until the filtrate was neutral. Finally, the LDH powders were dried in an oven at 60 °C before use.

### 3.3. Preparation of the SiF/LDH Nanocomposites

The foaming chemistry reactions for SiF relied on a dehydrogenative coupling reaction that occurred between PMHS and HO-PDMS for SiF, as well as an addition–cure reaction that occurred between Vi-PDMS and PMHS in the presence of a Pt catalyst for Vi-PDMS [53]. For the SiF/LDH nanocomposites, in detail, the reactive materials were divided and prepared in two parts. Part A was obtained by mixing Vi-PDMS (24.0 wt%), HO-PDMS (48.0 wt%), Pt catalyst (0.5 phr), and the LDHs (CoAl-LDH or NiAl-LDH) (1.0 phr) for 15 min under vigorous mechanical stirring (1500 rpm). Part B included Low-H PMHS (3.0 wt%), High-H PMHS (9.0 wt%), HO-PDMS (16.0 wt%), V4 (0.08 phr), and DI water (0.5 phr). After being prepared, the parts A and B were cooled in a refrigerator at 1–5 °C for 30 min, followed by a thorough mixing procedure with vigorous stirring (1500 rpm for 20 s). The as-prepared mixture was then quickly transferred into a polypropylene mold, where it was allowed to freely foam for 3 min. Finally, the SiF/LDH nanocomposite was put into an oven at 150 °C for 2 h for heat treatment before testing.

### 3.4. Characterizations

Fourier-transform infrared spectroscopy was utilized to analyze the chemical structures of CoAl-LDH and NiAl-LDH in a range from 4000 to 450 cm^−1^ (FTIR-ATR, Thermo Scientific, Nicolet iS50, Waltham, MA, USA). X-ray diffraction was used to confirm the phase compositions and crystallinity of CoAl-LDH and NiAl-LDH (XRD, Rigaku, Japan). X-ray photoelectron spectroscopy was adopted to analyze the chemical compositions of CoAl-LDH and NiAl-LDH (XPS, Thermo Scientific ESCALab 250Xi, Waltham, MA, USA). Inductively coupled plasma optical emission spectrometry was used to identify the metal element contents of the LDHs (ICP-OES, Thermo Fisher iCAP PRO, Waltham, MA, USA). The Brunauer–Emmett–Teller test was used to determine the specific surface areas and pore diameters of the LDHs (BET, Micromeritics ASAP 2460, Norcross, GA, USA). Scanning electron microscopy (SEM, Regulus8100, Japan), transmission electron microscopy (TEM, JEM-2100F, Tokyo, Japan), and atomic force microscopy (AFM, Bruker Dimension Icon, Germany) were used to analyze the microstructures of the LDHs. Ultra depth-of-field microscopy was used to study the microscopic surface topographies of the SiF/LDH nanocomposites (Leica DVM6M, Weztlar, Germany). The flame retardancy of the SiF/LDH nanocomposites was evaluated through vertical flame testing (VFT, CZF-3, Jiangning, China) and the limited oxygen index (LOI, HC-2C, Jiangning, China) according to GB/T 10707-2008 and ISO 4589, respectively. The fire behaviors of the SiF/LDH nanocomposites were evaluated with a cone calorimetric test with an external heat flux of 35.0 kW m^−2^, and the sample size was 100.0 × 100.0 × 10.0 mm^3^ (FTT, East Grinstead, UK). Scanning electron microscopy coupled with energy-dispersive X-ray spectroscopy was used to study the microscopic morphologies of the char layers and to determine the compositions of the char layers (SEM-EDS, Regulus8100, Tokyo, Japan). Thermogravimetric analysis was utilized to study the thermal decomposition of the LDHs and the SiF/LDH nanocomposites in air with a heating rate ramp of 20 °C min^−1^ from 40 °C to 800 °C (TGA, Perkin-Elmer STA 6000, Norwalk, CT, USA). According to GB/T 18942.2-2003, a universal testing machine was used to determine the cyclic compression properties of samples with a size 40 × 40 × 10 mm^3^ (INSTRON5967, Canton, MA, USA).

## 4. Conclusions

CoAl-LDH and NiAl-LDH were synthesized by using a co-precipitation method and were used in a flame-retardant SiF. The results of FTIR, XRD, XPS, and ICP-OES indicated that CO_3_^2−^-intercalated CoAl-LDH and NiAl-LDH were successfully prepared and characterized. According to the results of a Brunauer–Emmett–Teller, the specific surface areas of CoAl-LDH and NiAl-LDH were 51.47 and 174.57 m^2^ g^−1^, respectively. With only 1 phr of CoAl-LDH or NiAl-LDH, the SiF/LDH nanocomposites showed a higher LOI value (29.6%) compared with that of untreated SiF and passed the UL-94 V-1 rating. Meanwhile, the results of cone calorimetry showed that NiAl-LDH revealed a better smoke suppression effect in SiF than that of CoAl-LDH. This could be ascribed to the large specific surface area and good catalytic action of NiAl-LDH. The SEM-EDS analyses of the outer and inner surfaces of the char residues of the SiF/LDH nanocomposites indicated that both CoAl-LDH and NiAl-LDH participated in the formation of a protective char layer. In addition, the addition of CoAl-LDH and NiAl-LDH reduced the density of the silicon foam and increased the size of the cells, thereby slightly reducing the compressive strength of the silicone foam.

## Figures and Tables

**Table 1:** The corresponding data from the ICP-OES test.

Samples	Metal Element	Element Content (%)	Metal Elements’ Molar Ratios
CoAl-LDH	Co	42.71	Co: Al = 2.32
	Al	8.42	
NiAl-LDH	Ni	30.78	Ni: Al = 2.28
	Al	6.21	

**Table 2:** The results of the density, LOI value, and vertical flame testing.

Samples	Density (g cm^−3^)	LOI (vol%)	UL-94 (3.2 mm)			
			t_1_ (s)	t_2_ (s)	Rating	Dripping
SiF	0.28 ± 0.01	28.7	>30	-	NR	No
SiF/CoAl-LDH-1.0	0.27 ± 0.01	29.6	25 ± 2	1 ± 1	V-1	No
SiF/NiAl-LDH-1.0	0.26 ± 0.02	29.6	14 ± 3	8 ± 1	V-1	No

**Table 3:** Data on the SiF/LDH nanocomposites from the cone calorimeter tests.

Samples	pHRR (kW m^−2^)	t_p_ (s)	THR (MJ m^−2^)	TSP (m^2^)	FIGRA (kW m^−2^·s^−1^)	Residue (%)
SiF	112.8	130	43.0	9.5	0.87	76.0
SiF/CoAl-LDH-1.0	108.7	205	47.0	6.4	0.53	79.5
SiF/NiAl-LDH-1.0	104.3	190	47.1	5.2	0.55	79.9

**Table 4:** TGA data of the CoAl-LDH, NiAl-LDH, and SiF/LDH nanocomposites in an air atmosphere.

Samples	T_5%_ (°C)	T_max1_ (°C)	T_max2_ (°C)	T_max3_ (°C)	Char Residue at 800 °C (wt%)
CoAl-LDH	171	213	264	-	68.0
NiAl-LDH	141	184	317	-	63.7
SiF	380	367	480	562	50.1
SiF/CoAl-LDH-1.0	369	363	470	552	58.4
SiF/NiAl-LDH-1.0	368	366	495	557	50.9

## Data Availability

The data are available upon request.
